# Exploring the Application of Multi-Resonant Bands Terahertz Metamaterials in the Field of Carbohydrate Films Sensing

**DOI:** 10.3390/bios13060606

**Published:** 2023-06-02

**Authors:** Min Zhang, Guanxuan Guo, Yihan Xu, Zhibo Yao, Shoujun Zhang, Yuyue Yan, Zhen Tian

**Affiliations:** 1 Center for Terahertz Waves, Key Laboratory of Optoelectronics Information and Technology, Ministry of Education, Tianjin University, Tianjin 300072, China; 2 School of Precision Instrument and Optoelectronics Engineering, Tianjin University, Tianjin 300072, China; 3 Georgia Tech Shenzhen Institute (GTSI), Tianjin University, Shenzhen 518067, China

**Keywords:** metamaterial, multi-resonant bands, biomolecules, response sensitivity, recognition

## Abstract

Terahertz spectroscopy is a powerful tool for investigating the properties and states of biological matter. Here, a systematic investigation of the interaction of THz wave with “bright mode” resonators and “dark mode” resonators has been conducted, and a simple general principle of obtaining multiple resonant bands has been developed. By manipulating the number and positions of bright mode and dark mode resonant elements in metamaterials, we realized multi-resonant bands terahertz metamaterial structures with three electromagnetic-induced transparency in four-frequency bands. Different carbohydrates in the state of dried films were selected for detection, and the results showed that the multi-resonant bands metamaterial have high response sensitivity at the resonance frequency similar to the characteristic frequency of the biomolecule. Furthermore, by increasing the biomolecule mass in a specific frequency band, the frequency shift in glucose was found to be larger than that of maltose. The frequency shift in glucose in the fourth frequency band is larger than that of the second band, whereas maltose exhibits an opposing trend, thus enabling recognition of maltose and glucose. Our findings provide new insights into the design of functional multi-resonant bands metamaterials, as well as new strategies for developing multi-band metamaterial biosensing devices.

## 1. Introduction

Terahertz spectroscopy is an important means for investigating the characteristics of biomolecules, such as carbohydrates, proteins, and DNA [1,2,3,4,5,6], as their vibration and rotation frequencies fall within the terahertz frequency range, allowing them to exhibit certain absorption properties in this band [6,7,8,9]. Despite the potential of terahertz waves to measure biomolecules, their limited interaction makes it difficult to achieve high sensitivity detection of target biomolecules and distinguish multiple biomolecules [10]. In recent years, the emergence and advancement of terahertz metamaterials have enabled the detection of small biomolecules using terahertz waves [11,12,13,14]. Terahertz metamaterials are artificially constructed structures composed of periodic arrays of subwavelength units [15,16,17,18,19,20,21]. These metamaterials have been widely studied for their versatile and remarkable effects, such as circular dichroism, photoluminescence signal controllability, electromagnetically induced transparency, and near-perfect absorption [22,23,24,25,26,27,28]. Among them, metamaterials-based electromagnetically induced transparency (EIT) windows and resonant bands have been extensively investigated for their potential applications in biosensing and biomolecular detection due to their ability to provide multi-channel detection [29]. This ability is mainly attributed to the resonance coupling strength and resonance frequency of metamaterials [30]. A novel plasmonic THz metamaterial composed of metal strips coupled with parallel metal strip resonators was demonstrated, which is the first emulation of the single-EIT and double-resonant bands phenomena [31]. Metamaterials with EIT windows and resonant bands characteristics have been widely observed in cut wires [32], bilayer fish-scales, and split-ring resonators (SRRs) [33,34,35,36]. Subsequently, the pursuit of multiple EIT windows and multi-resonant bands has also attracted considerable attention [37,38], as terahertz waves assisted by metamaterials with multi-resonant bands have great potential to distinguish multiple biomolecules [39]. Currently, a few tunable plasmon metamaterials design with optically or electrically controlled regimes have been reported as potential solutions for realizing multiple transparent windows and multi-resonant bands [40,41,42,43]. For instance, Yu et al. discussed metamaterials featuring controllable photoluminescence intensities, which could help expand the biomedical applications of multi-resonant bands metamaterials [44]. Buono et al. examined nonlinear optics and structured light with multiple degrees of freedom, coupled via medium properties, highlighting the importance of metamaterial structures in shaping nonlinear process outcomes [45]. This offers a promising application for the design and performance enhancement of multi-resonant atomic structures. Cao et al. developed a tunable, reconfigurable extraordinary optical transmission (EOT) terahertz metamaterial, enabling reversible switching and tuning of the EOT effect [46]. Their design approach presents opportunities for creating multi-resonant bands metamaterial modulators. Although considerable effort has been devoted to various designs of metamaterials for realizing multi-EIT windows and multi-resonant bands, no regular control of multiple resonant peak quantities has been achieved [47,48,49,50]. Therefore, precisely controlling the number of transparency windows and multi-resonant bands remains a challenge. Additionally, the terahertz biosensing of the multi-resonant bands metamaterials has received relatively little attention.

In this study, multi-resonant bands terahertz metamaterial structures were designed. Through introducing the combination factor of (*m*, *n*) based on the “bright mode” resonators and “dark mode” resonators, we systematically explored the evolution from two to four resonant frequency bands. By manipulating the number and positions of resonant elements in the periodic unit cells, the number of resonant bands was adjusted, and a multi-resonant bands terahertz metamaterial with three EIT in four frequency bands was realized. Using this four-resonant-band metamaterial as a sensing platform, terahertz responses of maltose and glucose were studied. Due to the solvent water molecules and the complex many-body system with hydrogen bonds in biological solutions having a strong absorption of terahertz waves, the true signal of biomolecules is masked. It is difficult to achieve transmission-based concentration detection of the biomolecules in aqueous solution. Therefore, our research focused on biomolecular films in the state of crystalline. The terahertz metamaterial responses of maltose films and glucose films at different molecule masses were measured, as well as discrimination between these two biomolecules. It was found that the transmission peak of the four resonant bands metamaterials decreased with the biomolecule mass, and the resonance frequency redshifted with increasing biomolecule mass. Experiments revealed that the response sensitivity of this multi-resonant bands metamaterial could reach a high level at the resonance frequency similar to the characteristic frequency of the biomolecule. In addition, the frequency shift in glucose was always larger than that of maltose with increasing biomolecule mass in a specific frequency band. This four-resonant-band metamaterial and its associated study have great potential to support the design of multi-frequency terahertz sensors for quantitative detection and discrimination of multiple biological materials.

## 2. Materials and Methods

### 2.1. Preparation of the Materials, Biological-Solution and Biological-Films Sample

Glucose powder was purchased from Aladdin, while maltose powder was procured from Macklin Company. Deionized water was utilized to prepare solutions of varying concentrations of glucose and maltose. Various concentrations of glucose and maltose were dropped and dried on the surfaces of metamaterials to form films with different biomolecule masses.

### 2.2. Metamaterials Structure and Design

**Figure 1:**
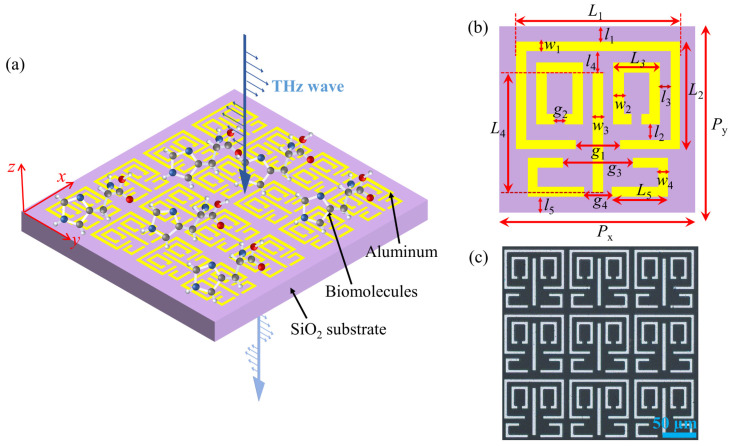
(**a**) Schematic drawing of the proposed multi-resonant bands terahertz metamaterial. The incidence direction and the electric field polarization direction of the THz waves are along the indicated z-axis (blue vertical arrows) and y-axis (blue horizontal arrows), respectively. The red arrow lines represent the spatial coordinate axes. (**b**) Periodic unit of the metamaterial. (**c**) Optical microscope image of the fabricated multi-resonant bands metamaterial. Geometrical parameters are *P*_x_ = *P*_y_ = 90 μm, *L*_1_ = 84 μm, *L*_2_ = 56 μm, *L*_3_ = 24 μm, *L*_4_ = 67 μm, *L*_5_ = 64 μm, *w*_1_ = *w*_2_ = *w*_4_ = 5 μm, *w*_3_ = 6 μm, *g*_1_ = 24 μm, *g*_2_ = 5 μm, *g*_3_ = 40 μm, *g*_4_ = 16 μm, *l*_1_ = 3 μm, *l*_2_ = 9 μm, *l*_3_ = 5 μm, *l*_4_ = 7 μm, and *l*_5_ = 5 μm.

Figure 1 shows the proposed multi-resonant bands terahertz metamaterial. The periodic unit of the metamaterial is made up of four resonant elements with different resonance frequencies. The size of the entire metamaterial array is 8.1 mm × 8.1 mm. The metamaterial structures are fabricated via photolithography on a 500 μm thick SiO_2_. The metamaterial structures are made of 200 nm-thick aluminum. The preparation processes are described in detail in the supplementary section (Figure S1). The optical microscope images of the fabricated metamaterials and four resonant elements are shown in Figure 1c and Figure S2.

CST Microwave Studio simulations show the transmission spectrum and the electric field distributions of the multi-resonant metamaterials. The SiO_2_ substrate is described by a dielectric constant of 3.58 with loss tangent delta of 0.02. The linearly polarized incident wave is perpendicular to the metamaterial surface. All measurements are carried out using a transmission-type THz time–domain spectroscopy system (THz-TDS). This system has a bandwidth of 0.1 THz to 2.0 THz by Fourier transform. A bare SiO_2_ substrate without metamaterials is used as the reference.

## 3. Results and Discussion

**Figure 2:**
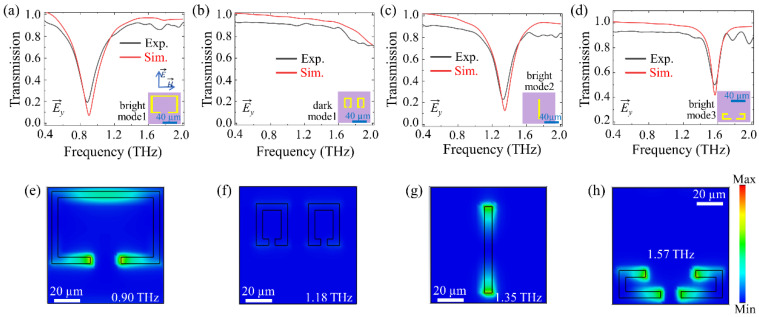
Experimental (black solid line) and simulated (red solid line) normalized transmission spectra of (**a**) the SRR only, (**b**) the TSRRs only, (**c**) the CW only, and (**d**) the LSRs only under *y*-orientation polarized incident radiation. Electric field enhancement for the (**e**) SRR at 0.90 THz, (**f**) TSRRs at 1.18 THz, (**g**) CW at 1.35 THz, and (**h**) LSRs at 1.57 THz under the *y*-orientation polarized incidence. The inset in (**a**–**d**) shows the unit cells of four resonant elements.

Figure 2 shows the simulated and experimental normalized transmission spectra of four resonant elements metamaterials: a SRR (Figure 2a), two small SRRs (TSRRs) (Figure 2b), a cut wire (CW) (Figure 2c), and L-split resonators (LSRs) (Figure 2d) under *y*-polarized incident waves. The normalized transmission spectra are defined as $\left|{\tilde{T}}_{\left(\omega \right)}\right|=\left|{\tilde{E}}_{S\left(\omega \right)}/{\tilde{E}}_{R\left(\omega \right)}\right|$, where ${\tilde{E}}_{S\left(\omega \right)}$ and ${\tilde{E}}_{R\left(\omega \right)}$ are Fourier transforms of the measured terahertz pulses of the sample and reference, respectively [51]. The SRR exhibits a transmission dip at 0.90 THz with a low-Q (Q = 3.75) resonance, referred to as the bright mode (Figure 2a,e). The TSRRs metamaterial displays a dark mode with almost no significant effect on the transmission when the gaps are perpendicular to the direction of the incident terahertz electric field (Figure 2b,f). Figure S3 presents the simulated transmission spectra and electric field distribution of TSRRs metamaterials under *x*- and *y*-polarized incidence. Under *x*-polarized incident waves, the two gaps parallel to the direction of the incident terahertz electric field, causing strong electric fields at both gap positions (Figure S3c). This corresponds to a transmission dip at 1.18 THz (Figure S3 red dashed line). The CW metamaterial has strong electric fields localized at the two ends, which correspond to a low-Q (Q = 5.9) dipole resonance at 1.35 THz, also known as the bright mode (Figure 2c,g). The LSRs exhibit a transmission dip at 1.57 THz, corresponding to a dipole resonance, which is also referred to as the bright mode (Figure 2d,h). The discrepancies between the experimental and simulated results are attributed to defects introduced in the metamaterials manufacturing process and the substrate dielectric losses that were neglected in the simulation [52]. In the following, we will design multi-resonant bands metamaterials with various combinations of SRR, TSRRs, CW, and LSRs, all under the *y*-orientation polarization of the incident wave.

**Figure 3:**
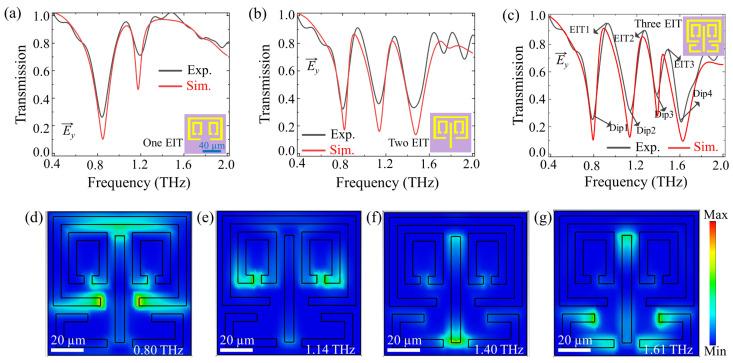
Experimental and simulation results for proposed multi-resonant bands metamaterials. Normalized transmission spectra for the (**a**) two-resonant bands metamaterial, (**b**) three-resonant bands metamaterial, and (**c**) four-resonant-band metamaterial under *y*-polarized incidences. (**d**–**g**) Electric field distribution at four resonant frequencies (Dip1, Dip2, Dip3, and Dip4) of the four-resonant-band metamaterial. The inset figures represent the unit cell of the corresponding metamaterials.

A two-resonant bands metamaterial, composed of an SRR and TSRRs, was first fabricated and measured (as shown in the inset of Figure 3a). This metamaterial exhibited an EIT with a transparency peak at 1.05 THz and two transmission dips (0.85 THz and 1.18 THz), as illustrated in Figure 3a. Here, a factor (*m*, *n*) was employed to represent the combination of the excitation resonance in the metamaterials, where *m* is the number of bright modes, and *n* is the number of dark modes. Therefore, the combination factor of this metamaterial was (*m*, *n*) = (1, 1). To further understand the EIT mechanism in two-resonant bands metamaterial, the electric field distribution and transmission spectra under *y*-polarized incident waves were simulated, as shown in Figure S4. The SRR is strongly excited by the incident wave at resonance 0.85 THz (Figure S4b). The near-field coupling between the bright mode SRR and the dark mode TSRRs excites the TSRRs resonance at 1.18 THz (Figure S4d). The coupling between SRR and TSRRs resonances leads to a distinct transparency window (Figure 3a and Figure S4a). At the transparency frequency of 1.05 THz, one can observe that the localized electric field is almost suppressed (Figure S4c). Further adjustments of the combination factor (*m*, *n*) are performed to realize metamaterials with more resonant bands. To create a three-resonant bands metamaterial, a CW (Figure 1e) was added to the unit cell of two-resonant bands metamaterial, as shown in the inset of Figure 3b. This resulted in a combination factor of (2, 1), with two bright modes and one dark mode. Three transmission dips (0.82 THz, 1.14 THz, and 1.47 THz) were observed, stemming from the resonances associated with each resonator element (Figure S5). Additionally, two transparency windows appeared at 0.9 THz and 1.26 THz due to the interaction between these three resonant modes (Figure 3b). The second transmission peak was attributed to the coupling between the CW and TSRRs.

Next, the four-resonant-band metamaterial, which consists of SRR, TSRRS, CW and LSRs, was fabricated and measured. As shown in the inset of Figure 3c, this unit cell has the bright mode factors of *m* = 3 and the dark mode factor of *n* = 1, giving a combination factor of (3, 1). Figure 3c displays the transmission spectra of the metamaterial, showing four transmission dips (0.80 THz, 1.14 THz, 1.40 THz, and 1.61 THz) and three transparency windows at 0.90 THz, 1.26 THz, and 1.47 THz. This behavior was further analyzed by simulating the electric field intensity distributions at the four transmission dips, as shown in Figure 3d–g. The first and second transmission dips were found to arise from the resonances associated with the SRR and TSRRs, respectively, with the first transparency window at 0.90 THz. The third resonant frequency of 1.40 THz displayed electric field concentration primarily on the CW ends, with the second transparency window stemming from the coupling between the TSRRs and CW. The fourth transmission dip was determined to arise from the resonances associated with the LSRs, with the third transparency window arising from the coupling between the CW and LSRs. The simulated transmission spectra (red line) showed good agreement with the measurements (black line), as shown in Figure 3.

The above experimental results prove that, by manipulating the number and positions of resonant elements in metamaterials, three EITs in four-resonant frequency bands can be achieved. The multi-resonant bands metamaterial sensors we will discuss next are all four-resonant-band metamaterial. We demonstrated the detection and recognition capability of the four-resonant-band metamaterial using maltose and glucose, which are essential saccharides with molecular weights of 342 g/mol and 180 g/mol. The volume of the maltose and glucose solution added on the surface of the metamaterials is 50 µL. Due to the strong absorption of terahertz waves by water molecules in the biological solution, it is difficult to achieve the transmission-based concentration analysis of the biological solution. Thus, we crystallized the biological solution at a temperature of 60 °C to form a dried film [53,54,55]. Images of the crystallized biological samples on metamaterials can be found in Figure S6. Although all biological solutions will be at 100% concentration after drying, the increased ratio of biomolecules in a fixed volume of the solution expanded the mass of the biomolecule, resulting in varying morphologies and thicknesses in the crystallized biological films. We represent the initial solution concentrations corresponding to the dried films in terms of the biomolecule mass. The biomolecule mass-dependent transmission spectra and frequency shifts discussed below, which are based on the metamaterial, reflect the differences in biological film morphology and thickness, thereby directly demonstrating the different biomolecule masses sensitivity of the metamaterial. The measurement was repeated nine times for each data point (*n* = 9), and the error bars of the experimental data represent the standard error of the mean.

**Figure 4:**
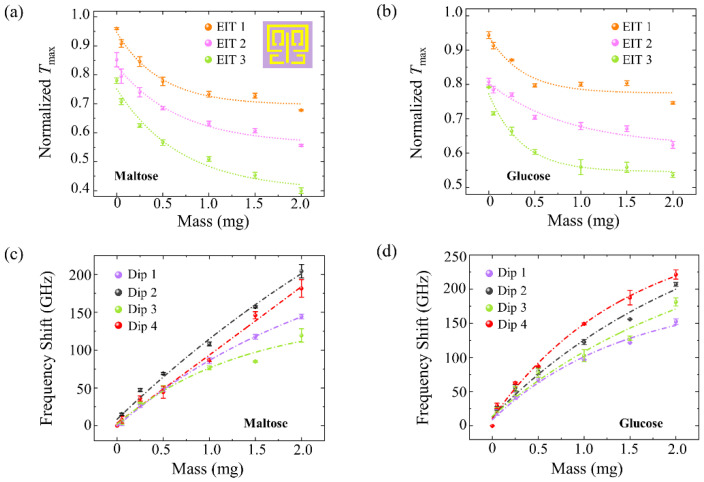
Experimental sensing results of linearly polarized illumination on the four-resonant-band metamaterial sensor. Normalized transmission peaks (*T*_max_) of three EIT windows (EIT1, EIT2, and EIT3) for different (**a**) maltose and (**b**) glucose molecule masses with their fitting results (shot dash-dot lines). The correlation coefficients of these fitting lines are R^2^_EIT1-maltose_ = 0.979, R^2^_EIT2-maltose_ = 0.977, R^2^_EIT3-maltose_ = 0.974, R^2^_EIT1-glucose_ = 0.922, R^2^_EIT2-glucose_ = 0.960, and R^2^_EIT3-glucose_ = 0.975, respectively. The frequency shift in (**c**) maltose and (**d**) glucose by metamaterial at four resonant frequencies (Dip1, Dip2, Dip3, and Dip4) with their fitting results (shot dash-dot lines). The correlation coefficients of these fitting lines are R^2^_Dip1-maltose_ = 0.999, R^2^_Dip2-maltose_ = 0.992, R^2^_Dip3-maltose_ = 0.977, R^2^_Dip4-maltose_ = 0.992, R^2^_Dip1-glucose_ = 0.986, R^2^_Dip2-glucose_ = 0.985, R^2^_Dip3-glucose_ = 0.966, and R^2^_Dip4-glucose_ = 0.993, respectively. The inset in (**a**) shows metamaterials unit cells of the four-resonant-band metamaterial.

The terahertz spectrum for maltose and glucose films at a molecule mass of 0.25 mg on bare SiO_2_ substrate did not show distinguishable features compared to the spectrum for a bare SiO_2_ wafer (Figure S7) due to the extremely small absorption cross-section of the molecule at the relevant frequency regime [10]. Figure S8 shows the normalized transmission spectra of the four-resonant-band metamaterial in different masses of maltose and glucose. The maximum values of the normalized experimental transmittance, *T*_max_, for glucose and maltose, are plotted in terms of the mass levels with exponential decay fittings (Figure 4a,b). The three EIT peaks of the four-resonant-band metamaterial decreased as the biomolecule mass increased due to the increasing dielectric loss of the biosensing target. The weakening of the transmission peak reduction caused by adding a biomolecule sample can be explained by the following interpretations. First, as a common property of surface waves, the intensity of the electric field decreases above the metamaterials. Second, this result may be due to the dielectric loss of the sensing target, which reduces the energy of the incident THz wave [53]. At low molecule masses, fewer dried crystalline biomolecules per unit area result in little influence on the incident wave energy and a larger decrease in transmission peak amplitude. At high molecule masses, there are more dried crystalline biomolecules per unit area, causing a reduction in the incident wave energy. The wave excites the localized surface plasmons of the metamaterial with weaker intensity, thus reducing the decrease in transmission peak amplitude.

The CST was used to simulate the sensing results of metamaterials with biological films, having different optical properties, as shown in Figure S11. The *T*_max_ at the EIT 2 window is analyzed as an example. Figure S11a–c demonstrates the influence of the dielectric loss tangent *δ* (0~0.7), refractive index (1.0~2.6), and thickness (1.0~3.4 µm) on metamaterial sensing. *T*_max_ experienced an exponential decrease with increasing loss tangent, as dielectric losses led to a decrease in electric field intensity on the metamaterials at 1.18 THz, as shown in Figure S11d–f. There were no obvious changes in *T*_max_ values with increasing refractive index. As the thickness of the biological film increases, the transmission peak gradually decreases. Therefore, the principal factor of metamaterial *T*_max_ reduction is the dielectric loss tangent and thickness of the sensing target. These simulation results coordinate well with the *T*_max_ nonlinear reduction in Figure 4a,b. At near-zero biomolecule masses, differences in the initial values (Figure 4a,b) arise from inherent experimental system errors, environmental moisture interference, and slight polarization direction deviations between the sample and linearly polarized incident wave upon multiple placements in the testing system. These factors collectively contribute to the observed transmission signal strength errors (more details are shown in Figures S9 and S10).

Although this change in EIT peaks reflects the different masses of maltose and glucose, it is difficult to distinguish between them. To address this, we studied the resonant frequency shift in these two biomolecule samples on the four-resonant-band metamaterial. As shown in Figure S12, the influences of the dielectric loss tangent *δ* (0~0.7), refractive index (1.0~2.6), and thickness (1.0~3.4 µm) on the frequency shift are first simulated. The frequency shift at the Dip 2 is analyzed as an example. There were no obvious changes in frequency shift with increasing loss tangent (Figure S12a). The frequency shift experienced an almost linear increase with the increment of refractive index and thickness (Figure S12b,c). Figure 4c,d shows the experimentally tested frequency shifts of the four resonance bands of the maltose and glucose with different biomolecule masses. It was observed that the sensitivity of these four resonant bands varied as the mass increased. The resonance frequencies are shifted toward a lower frequency with the value |f − shift_maltose_| and |f − shift_glucose_| as the biomolecule mass increases. The frequency shifts are almost linearly related to the biomolecule mass, which corresponds well with the trends shown in Figure S12. The frequency shift can be interpreted as the effect from the surrounding media, and the stronger shift with higher biomolecule mass is due to the thicker stacking and refractive index change in the biological film [10]. With an increasing maltose molecule mass, the frequency shift at Dip2 increased more promptly than at Dip1, Dip3, and Dip4 (Figure 4c), indicating a better response sensitivity for Dip2. The optimal frequency detection range of maltose was found to be 0.95 THz~1.14 THz. Similarly, with an increasing glucose molecule mass, the frequency shift at Dip4 increased more quickly than at Dip1, Dip2, and Dip3 (Figure 4d), indicating a better response sensitivity for Dip4. The best frequency detection range of glucose was determined to be 1.4 THz~1.61 THz. Our experimental and simulation findings reveal that, although this kind of metamaterial only provides a means to investigate the spectral response properties of maltose and glucose films in multiple windows and resonance bands and may not be able to quantitatively detect the concentration values of solution. It can differentiate between dried films with varying morphologies and thicknesses. In this way, it is considered to possess biomolecule mass sensitivity. The experimental results also demonstrated that there are only slight deviations between multiple repeated measurements, thereby substantiating the capability of this multi-resonant-band terahertz metamaterial to detect and distinguish two types of biomolecules within a high range of biomolecular masses.

Next, the maltose and glucose powders were formed into pellets with polyethylene (PE) at a mixing ratio of 1:1, respectively. The thickness of pellets is about 1 mm, the diameter is about 13 mm, and the mass is about 200 mg, as shown in Figure S13a. The transmission coefficients of maltose and glucose pellets were measured (Figure S13b,c) and found to have characteristic frequencies of 1.1 THz (Figure S13b) and 1.45 THz (Figure S13c), respectively. These results were consistent with the position of the two resonant bands (Dip2 and Dip4) with high response sensitivity for maltose and glucose, respectively. The experimental results demonstrated that this four-resonant-band metamaterial showed high response sensitivity at frequencies close to the characteristic frequency of the biomolecule. The design of these multi-resonant bands offers two distinct advantages. First, the most sensitive resonant frequencies of biomolecules can be found through this metamaterial with multi-resonant bands. Second, it facilitates the creation of metamaterial structures with more resonant bands tailored to match the characteristic frequencies of diverse biomolecular samples, addressing the needs of a wider array of biological specimens.

**Figure 5:**
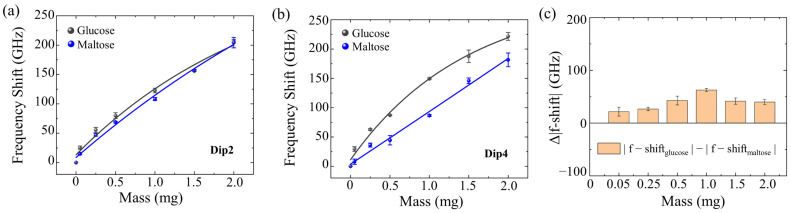
Maltose and glucose sensing using four-resonant-band metamaterial with different resonance dips. (**a**) Detection of maltose and glucose by metamaterials with a resonance dip2 at 1.14 THz. (**b**) Detection of maltose and glucose by metamaterials with a resonance dip4 at 1.61 THz. (**c**) Δ|f − shift| values for maltose and glucose on the metamaterial.

As shown in Figure 5a, with increasing biomolecule mass, the frequency shift in glucose is slightly larger than that of maltose at the frequency of Dip2, while the transmission coefficient of glucose pellets is significantly greater than that of maltose pellets (Figure S13). Likewise, Figure 5b demonstrates that, with increasing biomolecule mass, the frequency shift in glucose is significantly larger than that of maltose at Dip4, and the transmission coefficient of glucose pellets is smaller than that of maltose pellets (Figure S13). These results indicate that, compared to the transmission coefficient of the biological pellets, the change in dielectric constant due to increasing biomolecule mass is the primary factor for the distinguishment of different biological samples by the multi-resonant-band metamaterial. We measured the frequency shifts of glucose and maltose (|f − shift_glucose_| and |f − shift_maltose_| for glucose and maltose, respectively) on the four-resonant-band metamaterial and calculated the frequency shift difference at different biomolecule masses (Figure 5c), which is given by:

Δ|f − shift| = |f − shift_glucose_| − |f − shift_maltose_| (1)

Results demonstrated that the Δ|f − shift| was positive, indicating that the frequency shift in glucose was always larger than that of maltose at different masses. Then, it is easy to distinguish the glucose and maltose intuitively from the frequency shift difference Δ|f − shift| values.

**Figure 6:**
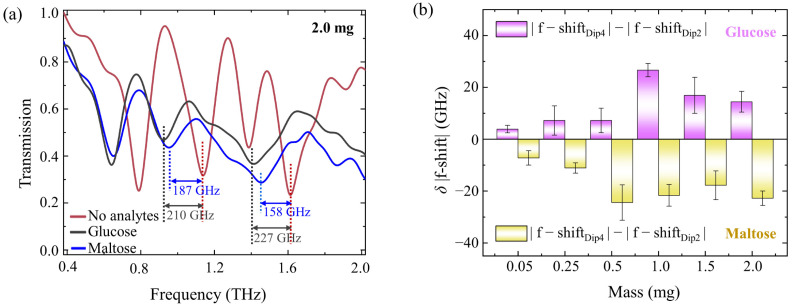
(**a**) Experimental normalized transmission spectra of maltose and glucose with the same biomolecule mass on the four-resonant-band metamaterial. (**b**) *δ*|f − shift| values for maltose and glucose on the metamaterial with different biomolecule masses.

It can be observed, from the normalized transmission spectra of the four-resonant-band metamaterial in a larger mass range of maltose and glucose, that there is a prominent difference in spectrum and frequency band positions between maltose and glucose films at the same mass (Figure 6a). Moreover, the frequency shift (Dip4) in glucose is significantly larger than Dip2, while the frequency shift (Dip4) in maltose is prominently smaller than Dip2. The frequency shift difference between Dip4 and Dip2 for the same biomolecule is defined as:

*δ*|f − shift| = |f − shift_Dip4_| − |f − shift_Dip2_| (2)

where; |f − shift_Dip4_| refers to the frequency shift at Dip4, and |f − shift_Dip2_| represents the frequency shift at Dip2. When the mass of the biomolecule is 2 mg, the *δ*|f − shift| of glucose is 17 GHz, exceeding zero, whereas, for maltose, the *δ*|f − shift| is −29 GHz, falling below zero. Subsequent calculations of frequency shift differences *δ*|f − shift| at varying masses for the same biomolecule are represented in Figure 6b. It can be seen that, as the mass of the biomolecule increases, *δ*|f − shift| for glucose remains invariably positive, while the maltose counterpart is consistently negative. These positive and negative values of *δ*|f − shift| further realized the qualitative and intuitive distinction of maltose and glucose molecules, highlighting the recognition capability of the four-resonant-band metamaterial toward these two types of biomolecule films. With increasing dielectric film thickness, the weakening of the excitation electric field intensity causes the frequency shift speed to be slightly slower (Figure S12c), thus resulting in the absolute value of *δ*|f − shift| first increasing, then gradually decreasing and becoming flat when increasing the biomolecule mass. Although the proposed strategy currently lacks the ability to explicitly distinguish different biomolecules in mixed biological solutions or mixed dried films through the spectra responses of multi-resonant bands metamaterials, it offers a promising outlook for the label-free, specific, and selective detecting of different biomolecules. Our future research will explore the integration of bio-enzymes, aiming to pioneer advancements in the specificity of terahertz metamaterial sensor selection, which will inspire us to perfect the booming THz biosensing technology.

## 4. Conclusions

In summary, this new four-resonant-band metamaterial has been designed by manipulating the number and positions of resonant elements in the periodic unit cell, and its biological detection capability has been demonstrated using maltose and glucose in the state of dried films as the analytes. The resonance frequencies redshift with increasing glucose and maltose molecule masses, as well as the response sensitivity, can reach a higher level at the resonance frequency, similar to the characteristic frequency of the biomolecule. For maltose, the optimal frequency detection range was 0.95 THz to 1.14 THz, and for glucose, the range was 1.4 THz to 1.6 THz. The frequency shift in glucose is always larger than that of maltose when increasing biomolecule masses. The differential between Dip4 and Dip2 frequency shifts of glucose invariably remained positive, whereas, for maltose, it invariably retained a negative value. Therefore, the positive or negative value of the frequency shift difference realized the distinction between maltose and glucose molecules. The results of this study indicate that this four-resonant-band metamaterial exhibits high response sensitivity and recognition of maltose and glucose, thus providing potential applications in multi-biomolecular unlabelled recognition biosensing in the terahertz regime.

## Data Availability

The data presented in this study are available upon request from the corresponding author.

## Supplementary Materials

The following supporting information can be downloaded at: https://www.mdpi.com/article/10.3390/bios13060606/s1, Figure S1: Schematic of the steps to prepare multi-resonant metamaterials; Figure S2: Optical microscope image of the fabricated four resonant elements; Figure S3: Simulated transmission spectra and electric field distribution of the TSRRs metamaterial; Figure S4: Simulated transmission spectra of the SRR, TSRRs and two-resonant bands metamaterials, and electric field distribution for the two-resonant bands metamaterial; Figure S5: Electric field distribution at three resonant frequencies of the three-resonant bands metamaterial; Figure S6: Picture of the biological sample on the four-resonant bands metamaterials sensor; Figure S7: Normalized terahertz spectra measured for a bare SiO_2_ wafer used as a substrate, 0.25 mg of maltose and 0.25 mg of glucose on the same SiO_2_ substrate; Figure S8: Experimental terahertz transmission spectra with different maltose masses and glucose masses of the four-resonant bands terahertz metamaterial; Figure S9: THz time-domain spectroscopy system tests of the time domain signal of for the same reference sample; Figure S10: Repeatability response of four-resonant bands metamaterials to three transparency windows and four resonant frequencies; Figure S11: Simulation results of normalized transmission peak of EIT 2 by four-resonant bands metamaterials for dielectric films with different loss tangent, refractive index and thickness, and electric field distribution (1.18 THz) of terahertz metamaterials with a dielectric film loss tangent of 0, 0.2, 0.4; Figure S12: Simulation results of frequency shift on the four-resonant bands metamaterial sensor at Dip 2 with loss tangent ranging from 0 to 0.7, refractive index ranging from 1.0 to 2.6, and thickness ranging from 1.0 to 3.4 µm; Figure S13: Optical camera image and the experimental terahertz transmission spectra of maltose pellets and glucose pellets.
