# Supplementary material for: Exploring the Application of Multi-Resonant Bands Terahertz Metamaterials in the Field of Carbohydrate Films Sensing
Source: Biosensors (Basel). 2023 Jun 2;13(6):606. doi: 10.3390/bios13060606 (PMC10296517; doi:10.3390/bios13060606)
Supplement: Supplementary file 1 [file biosensors-13-00606-s001.zip › biosensors-2325730-supplementary.pdf]

biosensors

MDPI

Supplementary Material

Exploring the Application of Multi-Resonant Bands Terahertz Metamaterials in the Field of Carbohydrate Films Sensing

Min Zhang [1,2], Guanxuan Guo [1,2], Yihan Xu [1,2], Zhibo Yao [1,2], Shoujun Zhang [1,2], Yuyue Yan [1,2] and Zhen Tian [1,2,3,*]

1 Center for Terahertz Waves and Key Laboratory of Optoelectronics Information and Technology (Ministry of Education), Tianjin University, Tianjin 300072, China;
2 School of Precision Instrument and Optoelectronics Engineering, Tianjin University, Tianjin 300072, China
3 Georgia Tech Shenzhen Institute (GTSI), Tianjin University, Shen Zhen 518067 , China
* Correspondence: tianzhen@tju.edu.cn.

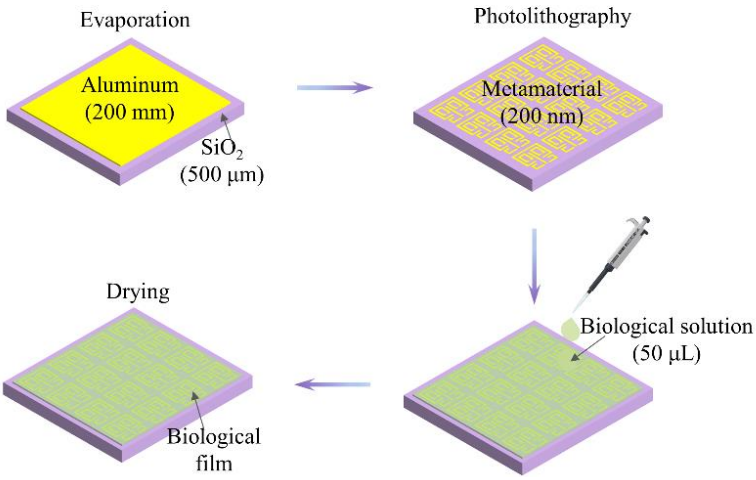

Figure S1. Schematic of the steps to prepare multi-resonant metamaterials.

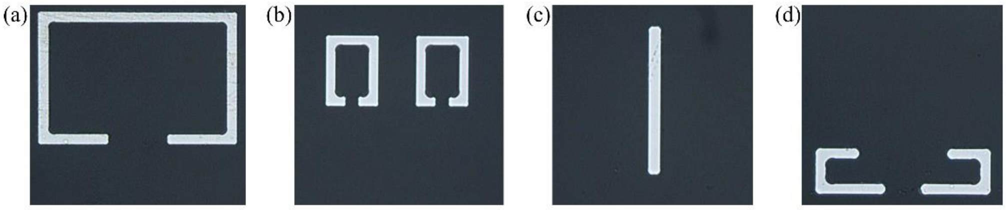

Figure S2. Optical microscope image of the fabricated four resonant elements. (a) Split-ring resonators (SRRs). (b) Two small SRRs (TSRRs). (c) Tut wire (CW). (d) L-split resonators (LSRs).

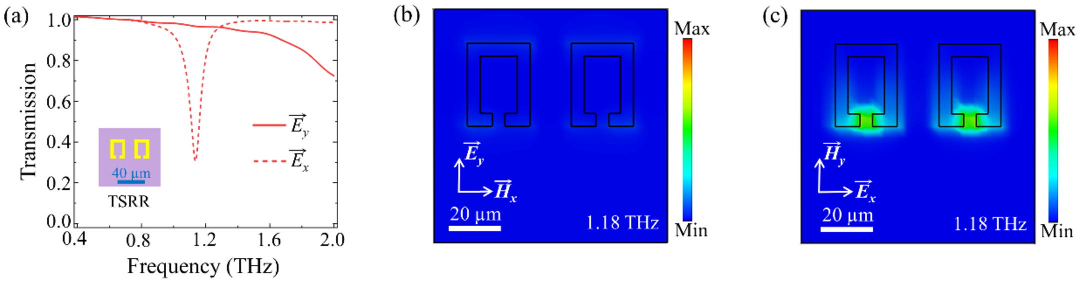

Figure S3. (a) Simulated transmission spectra of the TSRRs metamaterial under y-orientation (red solid line) and x-orientation (red dashed line) polarized incident radiation. Electric field distribution for the TSRRs at 1.18 THz under (b) y-orientation polarized incidence and (c) x-orientation polarized incidence. The inset figure represents the unit cell of the TSRRs metamaterial.

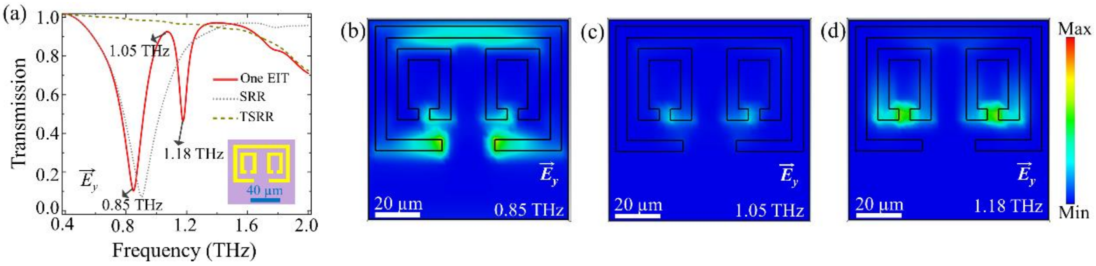

Figure S4. (a) Simulated transmission spectra of the SRR (black dotted line), TSRRs (green dashed line), and two-resonant bands metamaterials (red solid line) under y-orientation polarized incident radiation. Electric field distribution for the two-resonant bands metamaterial at (b) 0.85 THz, (c) 1.05 THz, and (d) 1.18 THz. The inset figure represents the unit cell of the two-resonant bands metamaterial.

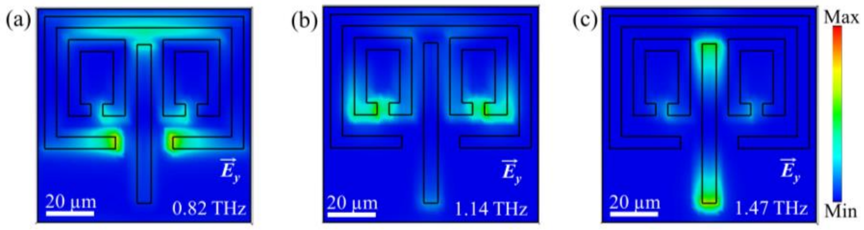

Figure S5. Electric field distribution at three resonant frequencies (a) 0.82 THz, (b) 1.14 THz, and (c) 1.47 THz of the three-resonant bands metamaterial.

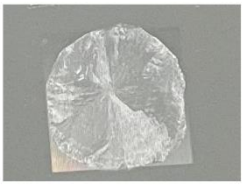

Figure S6. Picture of the biological sample on the four-resonant bands metamaterials sensor.

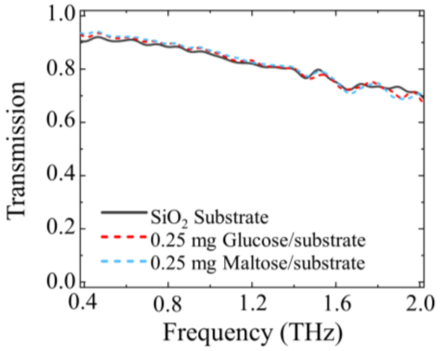

Figure S7. Normalized terahertz spectra measured for a bare SiO_2 wafer used as a substrate, 0.25 mg of maltose and 0.25 mg of glucose on the same SiO_2 substrate.

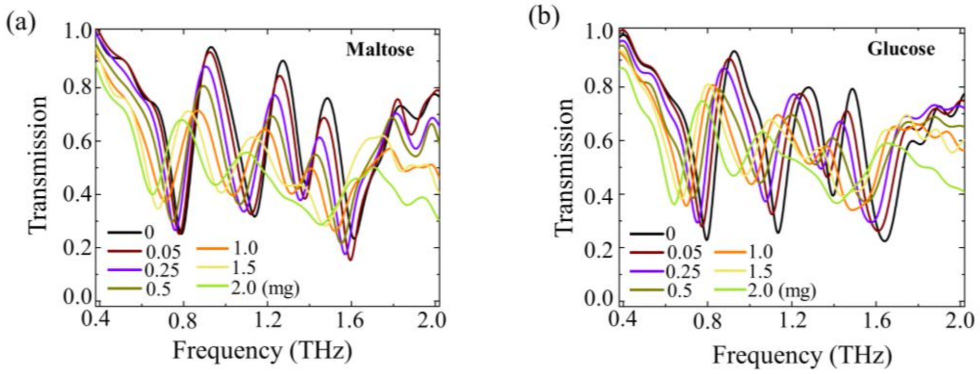

Figure S8. Experimental terahertz transmission spectra with different (a) maltose masses and (b) glucose masses of the four-resonant bands terahertz metamaterial under *y*- orientation polarized incident radiation.

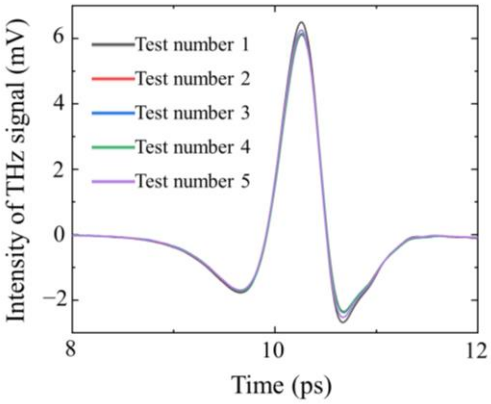

Figure S9. THz time-domain spectroscopy system (THz-TDS) tests of the time domain signal of for the same reference sample. Slight discrepancies exist among the five sets of tested transmission signals.

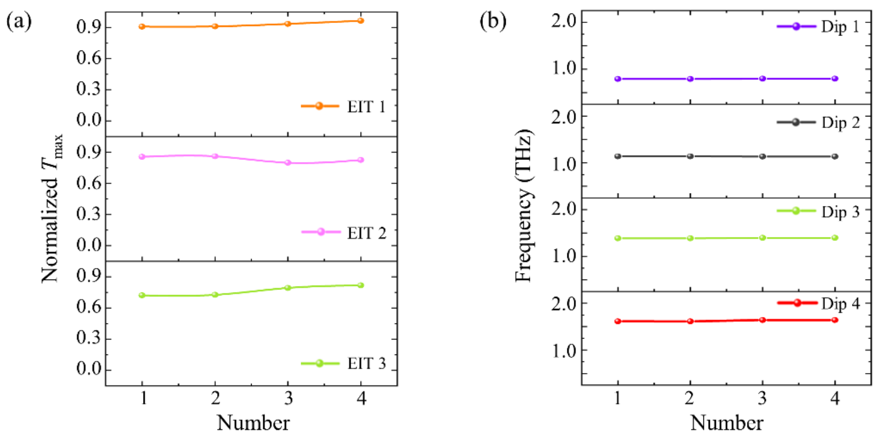

Figure S10. Repeatability response of four-resonant bands metamaterials to (a) three transparency windows (EIT1, EIT2, EIT3) and (b) four resonant frequencies (Dip1, Dip2, Dip3 and Dip4). The experiment observed minor discrepancies between the transmission signals from multiple tests.

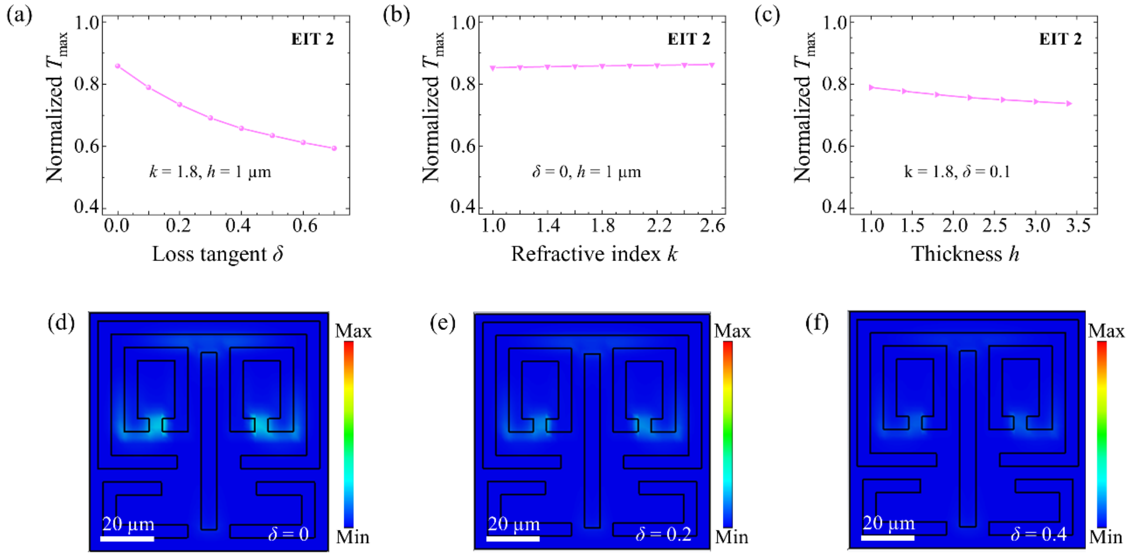

Figure S11. Simulation results of detection by four-resonant bands metamaterials for dielectric films with different loss tangent, refractive index and thickness. Normalized transmission peak of EIT 2 with (a) loss tangent ranging from 0 to 0.7, (b) refractive index ranging from 1.0 to 2.6, and (c) thickness ranging from 1.0 to 3.4 μm. (d-f) Electric field distribution (1.18 THz) of terahertz metamaterials with a dielectric film loss tangent of (d) δ = 0, (e) δ = 0.2, and (f) δ = 0.4. The refractive index of the dielectric film was 1.8.

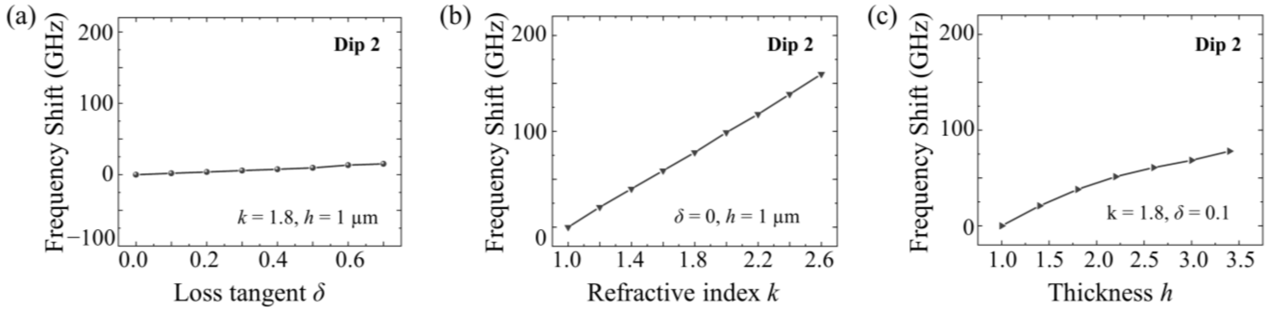

Figure S12. Simulation results of frequency shift on the four-resonant bands metamaterial sensor at Dip 2 with (a) loss tangent ranging from 0 to 0.7, (b) refractive index ranging from 1.0 to 2.6, and (c) thickness ranging from 1.0 to 3.4 μm.

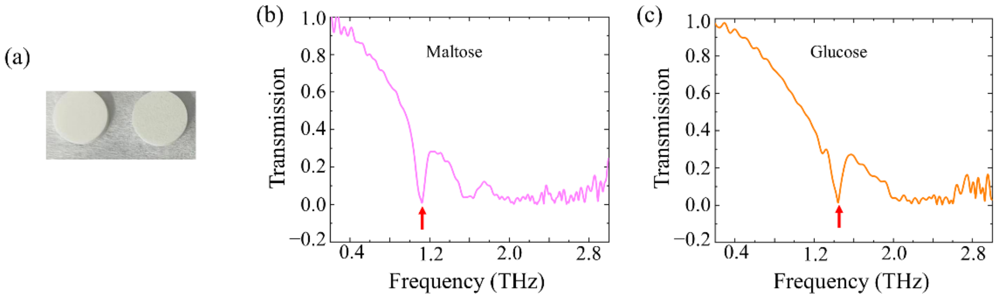

Figure S13. (a) Optical camera image of the maltose and glucose pellets. (b) Experimental terahertz transmission spectra of the (b) maltose pellets and (b) glucose pellets.
